# Exosomes from conditioned media of bone marrow-derived mesenchymal stem cells promote bone regeneration by enhancing angiogenesis

**DOI:** 10.1371/journal.pone.0225472

**Published:** 2019-11-21

**Authors:** Ryoko Takeuchi, Wataru Katagiri, Satoshi Endo, Tadaharu Kobayashi

**Affiliations:** Division of Reconstructive Surgery for Oral and Maxillofacial Region, Department of Tissue Regeneration and Reconstruction, Niigata University Graduate School of Medical and Dental Sciences, Niigata, Japan; Università degli Studi della Campania, ITALY

## Abstract

Growth factors in serum-free conditioned media from human bone marrow-derived mesenchymal stem cells (MSC-CM) are known to be effective in bone regeneration. However, the secretomes in MSC-CM that act as active ingredients for bone regeneration, as well as their mechanisms, remains unclear. Exosomes, components of MSC-CM, provide the recipient cells with genetic information and enhance the recipient cellular paracrine stimulation, which contributes to tissue regeneration. We hypothesized that MSC-CM-derived exosomes (MSC-Exo) promoted bone regeneration, and that angiogenesis was a key step. Here, we prepared an MSC-Exo group, MSC-CM group, and Exo-antiVEGF group (MSC-Exo with angiogenesis inhibitor), and examined the osteogenic and angiogenic potential in MSCs. Furthermore, we used a rat model of calvaria bone defect and implanted each sample to evaluate bone formation weekly, until week 4 after treatment. Results showed that MSC-Exo enhanced cellular migration and osteogenic and angiogenic gene expression in MSCs compared to that in other groups. *In vivo*, early bone formation by MSC-Exo was also confirmed. Two weeks after implantation, the newly formed bone area was 31.5 ± 6.5% in the MSC-Exo group while those in the control and Exo-antiVEGF groups were 15.4 ± 4.4% and 8.7 ± 1.1%, respectively. Four weeks after implantation, differences in the area between the MSC-Exo group and the Exo-antiVEGF or control groups were further broadened. Histologically, notable accumulation of osteoblast-like cells and vascular endothelial cells was observed in the MSC-Exo group; however, fewer cells were found in the Exo-antiVEGF and control groups.

In conclusion, MSC-Exo promoted bone regeneration during early stages, as well as enhanced angiogenesis. Considering the tissue regeneration with transplanted cells and their secretomes, this study suggests that exosomes might play an important role, especially in angiogenesis.

## Introduction

Stem cell-based therapy is a potent tool in regenerative medicine; mesenchymal stem cells (MSCs) are of special interest, given their multipotency, that is, the ability to differentiate into different cell lineages like osteoblasts [1, 2]. MSCs from multiple tissues have been reported to enhance bone regeneration in various models [3–6] and stem cell-based therapies have also been applied clinically to yield good results [7–9]. Although cell-based therapies including injection or transplantation of MSCs are promising strategies, some concerns remain, such as technical limitations and low survival rates of transplanted cells [10, 11]. Additionally, an increase in apoptosis after transplantation often triggers an immune response, resulting in worsening of the diseased condition or rejection of the transplanted cells [11, 12]. Furthermore, some studies reported that MSCs decrease in their differentiational ability as the donor age increases [13, 14].

Recent studies revealed that MSCs can contribute to tissue regeneration not only through their multipotency but also by stimulating the recipient cells via paracrine mechanisms [10, 15–17]. The paracrine effects are mediated by secretomes including cytokines and chemokines [16, 17]. As the secretomes from MSCs contain various factors exerting several biological effects, they are also expected to be applied clinically and provide novel strategies for regenerative medicine [18, 19]. Osugi *et al*. revealed that serum-free conditioned media from human bone marrow-derived MSCs (MSC-CM) contained numerous growth factors such as insulin growth factor-1 (IGF-1), vascular endothelial growth factor (VEGF), and transforming growth factor-β1 (TGF-β1), which accelerated bone regeneration [18, 20]. Katagiri *et al*. showed that VEGF acts as an effective growth factor for bone regeneration induced by MSC-CM through enhancing the migration of endogenous vascular endothelial and stem cells [21]; they also performed the first-in-human study of alveolar bone regeneration with good clinical outcomes [22]. Thus, understanding the molecular events that are regulated by secretomes will help contribute to a better understanding of tissue regeneration.

Angiogenesis is controlled by a series of released numerous factors whose balance dictates vessel formation and stability [23]. MSC-CM contain a number of angiogenic growth factors such as VEGF [18, 21, 24]; VEGF acts on vascular endothelial cells, and also enhances bone development by stimulating vascular endothelial cells [25, 26]. Osteogenesis is closely related to vascularization through cell-to-cell communication between vascular endothelial cells and osteoblasts, and sufficient vascularization is considerable for promoting osteogenesis [27]. Indeed, Kawai *et al*. revealed that MSC-CM contained abundant levels of VEGF, which enhanced angiogenesis and contributed to periodontal tissue regeneration [28]. Moreover, Katagiri *et al*. revealed that the addition of anti-VEGF antibody to MSC-CM reduced blood vessel formation and resulted in insufficient bone regeneration in a rat calvaria model [21]. However, the detailed underlying mechanisms in vascularized osteogenesis with cell-to-cell communication remain unknown.

Exosomes are nanovesicles (30 ~ 150 nm) with bilaminar membranes secreted from all cell types; they contain, amongst other molecules, microRNAs (miRNAs), which play important roles in controlling gene expression [29, 30]. Recent studies have focused on the role of communication between exosome-mediated cells in bone regeneration, and the therapeutic effect of exosomes has been thoroughly studied in various disease models [31–33]. Exosomes can provide recipient cells with genetic information, affecting their characteristics and paracrine factors, and resulting in tissue regeneration [33]. Exosomes are also contained in MSC-CM, and considered to be one of the key entities for clarifying the paracrine effects exerted by MSC-CM. However, how they specifically affect bone regeneration and angiogenesis remains unknown.

In this study, we investigated the effects of MSC-CM-derived exosomes (MSC-Exo) on bone regeneration, especially from the point of inducing angiogenesis.

## Materials and methods

All animal experiments were carried out in accordance with the protocols that were reviewed and approved by the Institutional Animal Care and Use Committee of Niigata University (approval No. SA00456). All efforts were made to minimize pain and distress during experiments. All animals were maintained on a 12-hours light/ dark cycle with free access to food and water. In order to confirm that no adverse events occurred, animals were visually checked every day during the experimental period. Animals were euthanized by overdose of sevoflurane at the end of the study.

### Preparation of MSC-CM

Human bone marrow-derived mesenchymal stem cells (hMSCs) were purchased from Lonza Inc. (Walkersville, MD, USA) and cultured in Dulbecco’s modified Eagle medium (DMEM; Gibco, Rockville, MD, USA) with 10% fetal bovine serum (FBS; Biowest, Nuaillé, France). Cells were maintained at 37°C in a humidified 5% CO_2_ incubator, and media was refreshed every 3 days. The cells were trypsinized and subcultured, and hMSCs at the 3rd to 6th passages were used for experiments.

When hMSCs reached 80% confluence, the medium was replaced by serum-free DMEM (DMEM(-)). The cell-cultured conditioned medium was collected after an additional 48 hours of incubation and filtered through a 0.22 μm filter sterilizer. The collected medium was defined as hMSC cultured conditioned medium (MSC-CM) and was stored at 4 or -80°C before use in the subsequent experiments.

### Isolation and identification of MSC-Exo

For MSC-Exo isolation, exosomes-depleted FBS was obtained by subjecting FBS to ultracentrifugation at 100 000 *g* for 16 hours to remove exosomes. Then, hMSCs were cultured in DMEM with 10% exosome-depleted FBS. MSC-CM was ultra-centrifuged at 100 000 *g* for 70 minutes. The supernatants were transferred to PBS and further ultra-centrifuged for 70 minutes. The pelleted extracellular vesicles (MSC-Exo) were resuspended and stored at -80°C. All procedures were performed at 4°C.

Purified MSC-Exo morphologies were observed using a transmission electron microscope (TEM; JEM-1400 Plus, JEOL Ltd., Tokyo, Japan). A drop of MSC-Exo suspension was pipetted onto a carbon-coated grid which had been treated to be hydrophilic. After a few minutes of standing, the excess fluid was removed, and then the sample was negative-stained with uranyl acetate for a few minutes and analyzed by TEM.

The common exosomal surface markers CD9, CD63, and CD81 were measured by western blotting. MSC-Exo, MSC-CM, and MSC lysates were diluted at a ratio of 1:1 with protein loading buffer (2×) and separated in a 10–20% gradient sodium dodecyl sulfate-polyacrylamide gel electrophoresis (SDS-PAGE) gel. After electrophoresis, proteins were transferred to polyvinylidene difluoride membranes, and blocked with Blocking One (Nacalai Tesque, Inc., Kyoto, Japan). The membranes were incubated overnight with the primary antibody CD9 (1:200; sc-59140, Santa Cruz Biotechnology, TX, USA), CD63 (1:200; sc-5275, Santa Cruz Biotechnology), or CD81 (1:400; sc-166029, Santa Cruz Biotechnology) at 4°C. After washing with tris-buffered saline containing Tween 20 (TBS-T) three times, the membranes were incubated for 1 hour with goat anti-mouse horseradish peroxidase (HRP) secondary antibody (1:3000) at room temperature. The bands were detected using ECL Select Western Blotting Detection Reagent (GE Healthcare UK Ltd., Buckinghamshire, UK) and images were captured using an Image Quant LAS 4000 mini (GE Healthcare).

The size distribution and particle concentration of MSC-Exo were determined using nanoparticle tracking analysis (NanoSight NS300, Malvern Panalytical Ltd., Worcestershire, UK).

### Enzyme-linked immunosorbent assay (ELISA)

The concentration of exosomes in MSC-CM was measured using Human CD9/CD63 Exosome ELISA Kit (Cosmo Bio Co., Ltd., Tokyo, Japan) according to the manufacturer’s instructions. Also, the concentrations of VEGF in cultured conditioned medium of 80% confluent hMSCs with MSC-Exo (5 μg/mL) + DMEM(-) (Exo-CM) or with MSC-CM for 48 hours were measured using Human VEGF Quantikine ELISA Kit (R&D Systems, Inc., MN, USA) according to the manufacturer’s instructions. The concentration of each sample was determined by measurement of optical density at 450 nm with a microplate spectrophotometer (Multiskan FC, Thermo Fisher Scientific, Inc., MA, USA).

### Migration assay

The hMSCs (5 × 10^5^ cells/cm^2^) in DMEM(-) were dispersed within the upper chamber of transwell dishes with 8 μm polycarbonate membranes (Corning Inc., NY, USA). MSC-Exo (5 μg/mL) + DMEM(-) (MSC-Exo), MSC-Exo (5 μg/mL) + anti-VEGFA antibody (100 ng/mL; ab39250, Abcam PLC, Cambridge, UK) + DMEM(-) (Exo-antiVEGF), MSC-CM, or DMEM(-) were placed on the lower chamber. After 48 hours of incubation, the surface of the membrane was rinsed with phosphate-buffered saline (PBS) and wiped with a cotton bud. The membrane was then stained with hematoxylin and the total number of cells that migrated was counted.

### Alizarin red S staining

The hMSCs were cultured with MSC-Exo (5 μg/mL) + DMEM(-) (MSC-Exo), MSC-Exo (5 μg/mL) + anti-VEGFA antibody (100 ng/mL) + DMEM(-) (Exo-antiVEGF), MSC-CM, or DMEM with 5% FBS for 14 days. Each medium was replaced every 3 days. After 14 days, hMSCs were washed twice in PBS and fixed in 10% neutral formalin for 15 minutes. The cells were stained with alizarin red S (FUJIFILM Wako Pure Chemical Co., Osaka, Japan) for 30 minutes. The area of the mineralized matrix depositions was observed by light microscopy (ECLIPSE TS100, Nikon Co., Tokyo, Japan).

### RNA extraction and quantitative reverse transcriptase-polymerase chain reaction (qRT-PCR)

The hMSCs were cultured with MSC-Exo (5 μg/mL) + DMEM(-) (MSC-Exo), MSC-Exo (5 μg/mL) + anti-VEGFA antibody (100 ng/mL) + DMEM(-) (Exo-antiVEGF), MSC-CM, or DMEM(-) for 48 hours. Total RNA was extracted using an RNeasy Mini kit (QIAGEN N.V., Venlo, Netherlands) and treated with RNase-free DNase set (QIAGEN) to remove potential genomic DNA contamination, and then reverse-transcribed into cDNA using PrimeScript RT Master Mix (TaKaRa Bio Inc., Shiga, Japan) according to the manufacturer’s instructions. The qRT-PCR analysis was performed using TB Green Premix Ex Taq II (TaKaRa Bio) in combination with Thermal Cycler Dice Real Time System III (TaKaRa Bio). The sequences of specific primers of osteogenesis-related genes (alkaline phosphatase (*ALP*), type I collagen (*COL I*), osteocalcin (*OCN*), and osteopontin (*OPN*)) and angiogenesis-related genes (vascular endothelial growth factor (*VEGF*), angiopoietin 1 (*ANG1*), and angiopoietin 2 (*ANG2*)) are listed in Table 1. Glyceraldehyde-3-phosphate dehydrogenase (*GAPDH*) was used as an internal control for PCR amplification. The 2–ΔΔCt method was used to calculate relative expression levels.

**Table 1:** Primer sequences used for qRT-PCR.

Gene		Sequence	Accession no.
*ALP*	F	5'-GCCATTGGCACCTGCCTTAC-3’	NM_000478.5
	R	5'-AGCTCCAGGGCATATTTCAGTGTC-3’	
*COL1*	F	5’-CCCGGGTTTCAGAGACAACTTC-3’	NM_000088.3
	R	5’-TCCACATGCTTTATTCCAGCAATC-3’	
*OCN*	F	5’-CATGAGAGCCCTCACACTCCT-3’	NM_199173.5
	R	5’-CACCTTTGCTGGACTCTGCAC-3’	
*OPN*	F	5’-ACACATATGATGGCCGAGGTGA-3’	NM_000582.2
	R	5’-GTGTGAGGTGATGTCCTCGTCTGTA-3’	
*VEGF*	F	5’-TCACAGGTACAGGGATGAGGACAC-3’	NM_001025366.2
	R	5’-CAAAGCACAGCAATGTCCTGAAG-3’	
*ANG1*	F	5’-TCAACATCTGGAACATGTGATGGA-3’	NM_001146.4
	R	5’-CTGAACTGCATTCTGCTGTATCTGG-3’	
*ANG2*	F	5’-GCTGAAGTATTCAAATCAGGACACA-3’	NM_001118887.1
	R	5’-ATCAACGCTGCCATCCTCA-3’	
*GAPDH*	F	5’-AGGCTAGCTGGCCCGATTTC-3’	NM_001256799.2
	R	5’-TGGCAACAATATCCACTTTACCAGA-3’	

### Rat calvaria bone defect model

To evaluate the bone regeneration ability of MSC-Exo, critical-sized defects in the rat parietal bones were created. For this study, 10-week-old male Wistar rats were anesthetized by intraperitoneal injection of 20 mg/kg pentobarbital. After shaving the parietal region of rats, a mucosal periosteal incision was made on the line connecting the two ear bases and peeled forward from the incision to clearly show the sagittal suture of the parietal bone. Two defects of 5 mm in diameter were carefully made in the calvarial bone using a trephine drill (Micro Tech. Corp., Tokyo, Japan), while avoiding damage to the dura mater. The defects were rinsed with PBS to remove the bone debris. Atelocollagen sponges (Terudermis®, Olympus Terumo Biomaterials Corp., Tokyo, Japan) were used as a scaffold, and were soaked with MSC-Exo (30 μg), MSC-Exo (30 μg) + anti-VEGFA antibody (1 μg), MSC-CM, or PBS. Sponges were implanted into the defects in calvarial bones. Finally, the mucoperiosteal flaps were sutured with 4–0 nylon thread. Two or four weeks after implantation, the specimens were harvested.

The rats were randomly divided into four groups (n = 6 in each group) and each of the experimental solutions soaked with atelocollagen sponges were implanted. The experimental groups were as follows: MSC-Exo group, Exo-antiVEGF group, MSC-CM group, PBS group, or defect group (defect only).

### Microcomputed tomography (Micro-CT) analysis

Samples from all groups were examined using a micro-CT system (CosmoScan Gx, Rigaku Co., Tokyo, Japan). The calvarial bones were observed by micro CT every week after implantation until harvesting specimens 2 or 4 weeks later. Rats were scanned under anesthesia by intraperitoneal injection of 4% chloral hydrate. Three-dimensional (3D) images were reconstructed using Analyze 12.0 software (AnalyzeDirect Inc., KS, USA). The newly formed bone area was evaluated as a percentage of the total bone defect.

### Histological analysis

Samples from all groups were harvested 2 or 4 weeks after implantation. The specimens were fixed in 10% neutral formalin, and then decalcified with 10% EDTA (pH 7.4). After 4 weeks of decalcification, samples were embedded in paraffin and sectioned at 4 μm thickness in the coronal plane using a microtome (REM-710, YAMATO KOHKI Industrial Co., Ltd., Saitama, Japan). Sections were dewaxed, rehydrated, stained with hematoxylin-eosin, and analyzed using a light microscope (FX630, OLYMPUS Co., Tokyo, Japan) and FLVFS-LS software (OLYMPUS Co.).

### Immunohistochemical analysis

Immunohistochemical staining was performed for osteocalcin (OCN) (1:1000; ABOC-5021, Haematologic Technologies Inc., VT, USA) to evaluate osteogenesis, while staining for VEGF (1:1000; ab39250, Abcam) was performed to detect angiogenesis. The sections were rehydrated, subjected to antigen retrieval using citrate buffer (pH 6.0) for 10 minutes at 121°C, and blocked for endogenous peroxidase with 0.3% H_2_O_2_ in methanol and incubated for 30 minutes. After washing with PBS, the sections were blocked for non-specific binding using 5% skim milk solution for 1 hour at room temperature, and then incubated with the primary antibody overnight at 4°C. Subsequently, the sections were reacted with EnVision Plus (Dako, CA, USA) for 1 hour and developed with 3,3′-Diaminobenzidine (DAB) solution. Hematoxylin counterstaining was performed following the DAB reaction.

Immunofluorescence staining for CD44 (1:2000; ab189524, Abcam) was performed to measure rat stem cells, and CD31 (1:50; ab28364, Abcam) was performed to measure new blood vessels in each group. Briefly, the sections were rehydrated, subjected to antigen retrieval using citrate buffer (pH 6.0), and blocked with casein solution. The sections were incubated with the primary antibody overnight at 4°C, and subsequently incubated with the secondary antibody. Alexa Fluor 488 (1:1000; ab150077, Abcam) and Alexa Fluor 647 (1:1000; ab150079, Abcam) were used as secondary antibodies. The sections were incubated with DAPI solution (Abcam) to stain the nucleus and observed with a fluorescence microscope (Axioplan 2, Carl Zeiss AG, Oberkochen, Germany).

### Statistical analysis

All data were analyzed as mean ± standard deviation (SD). Comparisons between experimental groups and control groups were analyzed by means of Tukey’s honestly significant difference test. The differences were considered statistically significant when *p* < 0.05.

## Results

### Isolation and characterization of MSC-Exo

**Fig 1:**
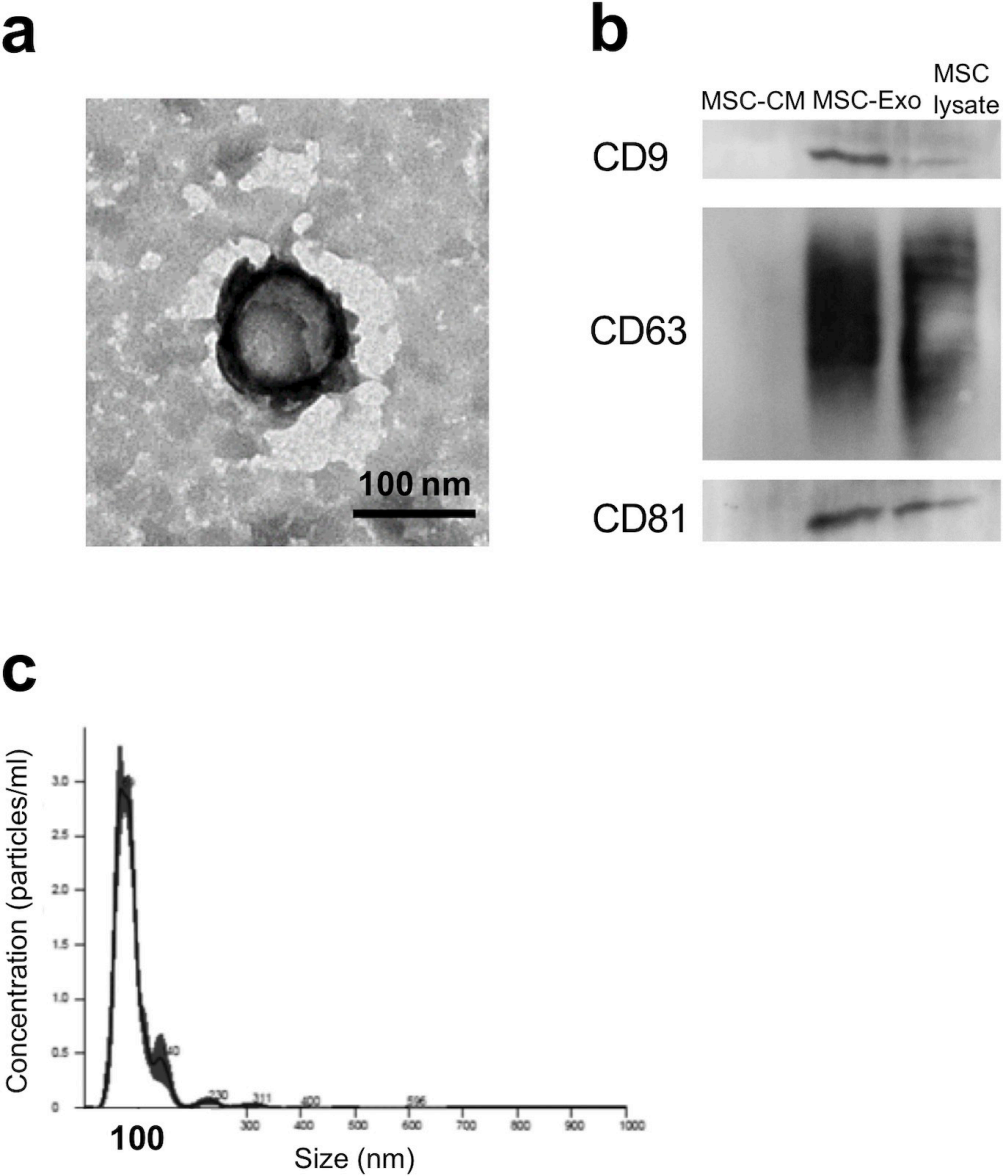
Characterization of MSC-Exo. (a) Morphology observed by TEM. Scale bar: 100 μm. (b) Western blot analysis of the exosomal surface markers CD9, CD63, and CD81. (c) The size distribution and particle concentration of MSC-Exo measured using nanoparticle tracking analysis.

The exosomes were isolated from the cultured conditioned media of hMSCs by ultracentrifugation. In the purified materials, typical exosome structures were observed by TEM, whereby a large majority of particles exhibited a round-shaped morphology (Fig 1A). Expression of the characteristic surface markers on isolated particles was confirmed, and CD9, CD63, and CD81 were detected by western blotting; these biomarkers are conserved in exosomes and widely used for testing exosomes (Fig 1B). Moreover, the size distribution and particle concentration of MSC-Exo were determined by nanoparticle tracking analysis. The exosomes had a narrow size distribution, which peaked at 80–100 mm, indicating that these particles were exosomes (Fig 1C).

### The presence of MSC-Exo in MSC-CM, and VEGF concentrations in Exo-CM and MSC-CM

The concentration of exosome in MSC-CM was determined. MSC-CM contained CD9 and CD63 at a total concentration of 42.6 ± 1.5 pg/mL. The concentration of VEGF in Exo-CM was 1140.3 ± 90.4 pg/mL, whereas the concentration of VEGF in MSC-CM was 1219.9 ± 126.6 pg/mL, supporting that Exo-CM contains equivalent amounts of VEGF when compared to MSC-CM.

### MSC-Exo increased hMSC migration

**Fig 2:**
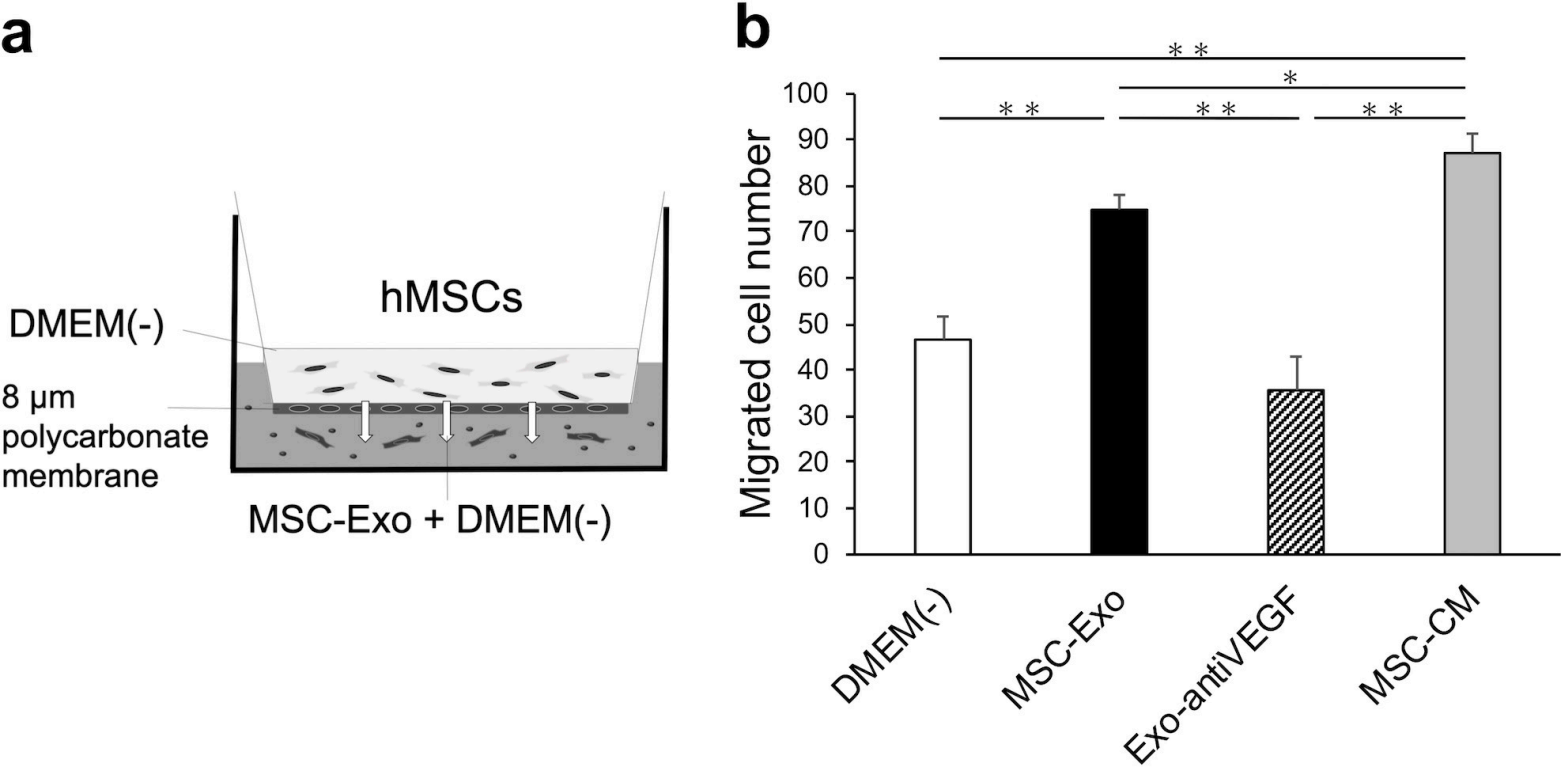
The effect of MSC-Exo on hMSC migration. (a) The experimental design of the transwell chamber assay. (b) The number of hMSCs that migrated in the DMEM(-), MSC-Exo, Exo-antiVEGF, and MSC-CM groups. (n = 6 per group. *, *p* < 0.05; **, *p* < 0.01).

The effect of MSC-Exo on hMSC migration was analyzed by transwell chamber assay (Fig 2A). After 48 hours of cell culture with each medium, the cells that migrated were counted. The number of migrated hMSCs was significantly higher in the MSC-Exo group (74.8 ± 3.2) compared to that in the DMEM(-) group (46.7 ± 5.0) (*p* <0.01). The number of migrated hMSCs in the MSC-CM group (87.1 ± 4.2) was also significantly higher compared to that in the DMEM(-) group (46.7 ± 5.0) (*p* < 0.01). Thus, the number of hMSCs that migrated was 62% higher in the MSC-Exo group compared to the control group. On the other hand, the number of migrated cells was significantly lower in the Exo-antiVEGF group (35.6 ± 7.3) compared to that in the MSC-Exo group and the MSC-CM group (*p* < 0.01) (Fig 2B). The number of migrated cells was clearly visible in the membrane images (S1 Fig).

### MSC-Exo induced mineral deposition in hMSCs

**Fig 3:**
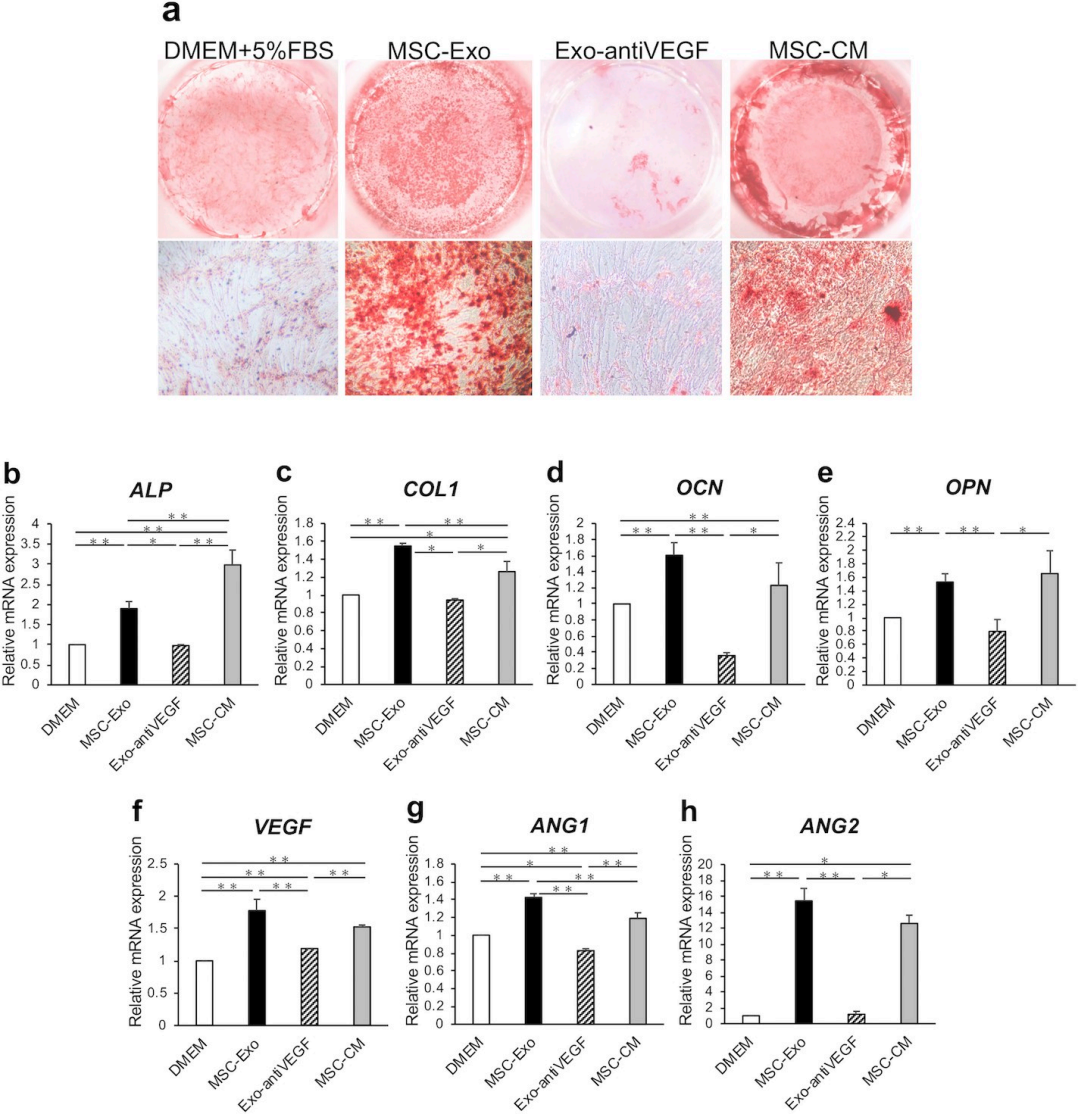
The osteogenesis and angiogenesis effects of MSC-Exo on hMSCs. (a) Views of Alizarin red S stained hMSCs incubated with MSC-Exo, Exo-antiVEGF, MSC-CM, and DMEM with 5% FBS for 14 days. (b-h) mRNA expression of osteogenesis and angiogenesis-related genes *ALP* (b), *COL1* (c), *OCN* (d), *OPN* (e), *VEGF* (f), *ANG1* (g), and *ANG2* (h) treated with MSC-Exo, Exo-antiVEGF, MSC-CM, and DMEM(-). (n = 5 per group. *, *p* < 0.05; **, *p* < 0.01).

To determine the effect of MSC-Exo on hMSC calcification, mineral deposition in hMSCs were observed following alizarin red S staining. The mineral deposition in the MSC-Exo group was markedly enhanced compared with that in the Exo-antiVEGF group (Fig 3A). These results indicate that MSC-Exo enhanced the differentiation potential of hMSCs into osteoblasts. However, in the Exo-antiVEGF group, the mineral deposition was insufficient, suggesting that osteoblast differentiation ability might be poor.

### MSC-Exo enhanced osteogenesis and angiogenesis-related gene expression in hMSCs

The transcript expression levels of *COL I*, *ALP*, *OCN*, and *OPN* were significantly upregulated in hMSCs cultured with MSC-Exo compared with those cultured with DMEM(-) and Exo-antiVEGF (*p* < 0.01) (Fig 3B–3E). The expression of *VEGF*, *ANG1*, and *ANG2* were also significantly upregulated in hMSCs cultured with MSC-Exo compared to that with DMEM(-) and Exo-antiVEGF (*p* < 0.01) (Fig 3F–3H). These results indicate that MSC-Exo enhanced the osteogenic and angiogenic potentials of hMSCs *in vitro*.

### MSC-Exo promoted bone formation *in vivo*

**Fig 4:**
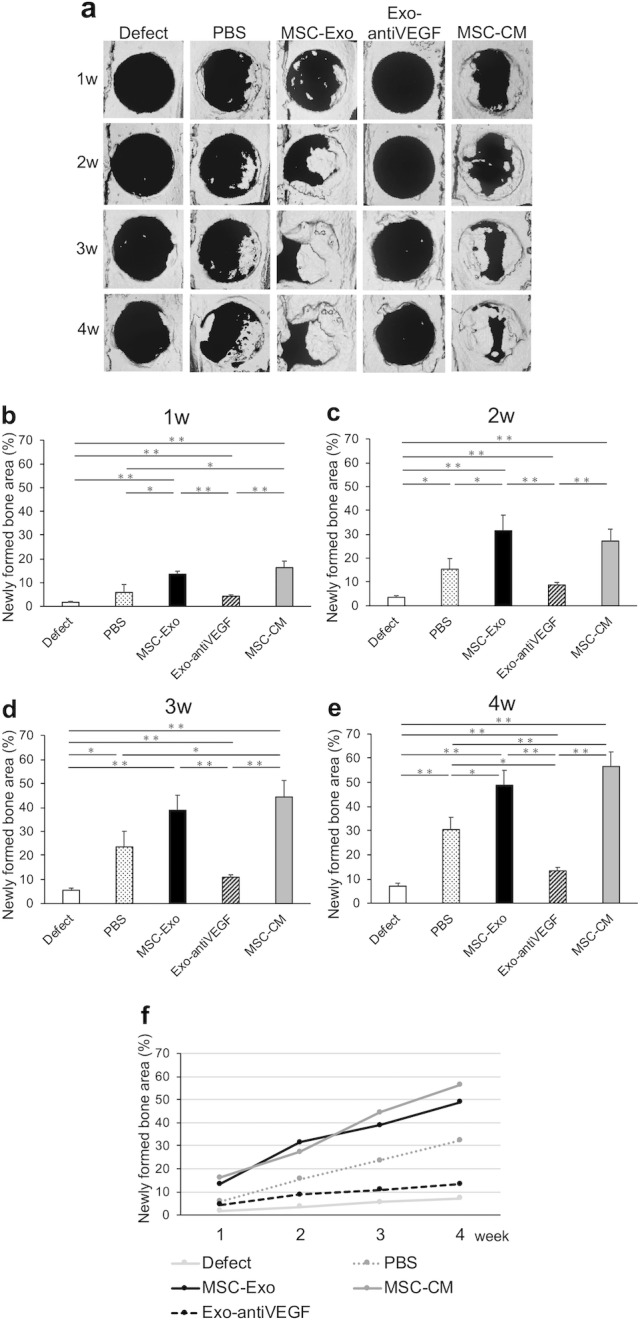
The effects of bone formation of MSC-Exo measured by Micro-CT *in vivo*. Rats were scanned every week until week 4 after implantation. (a) 3D reconstruction images at superficial side of rat calvarial bone defects implanted with PBS, MSC-Exo, Exo-antiVEGF, MSC-CM, and no implants (defect only). The new bones started to form from 2 weeks after implantation in the PBS, MSC-Exo, and MSC-CM groups. Four weeks after implantation, the newly formed bone was covering half of the defect in the MSC-Exo group. (b-e) Quantitative analysis of the new bone area in each group over a period of 1 to 4 weeks after implantation. (f) The time course of the newly formed bone area in each group.

The newly formed bone in the rat calvarial bone defect was evaluated with micro-CT scanning every week after implantation. Three-dimensional micro-CT reconstruction images revealed the morphology of the newly formed bone (Fig 4A). Quantitative analysis of the newly formed bone area was performed as an evaluation of percentage of the total graft area (Fig 4B–3F). One week after implantation, the newly formed bone areas in the defect (1.7 ± 0.4%), PBS (5.9 ± 3.3%), and Exo-antiVEGF (4.3 ± 0.6%) groups were less than 10% of the defect area; in contrast, the areas in the MSC-Exo (13.5 ± 1.3%) and MSC-CM (16.3 ± 2.8%) groups were significantly higher and over 10%.

The new trabecular bone started to form from 2 weeks after implantation in the MSC-Exo and MSC-CM groups. Two and three weeks after implantation, the newly formed bone areas in the MSC-Exo (2 w: 31.5 ± 6.5%, 3 w: 38.8 ± 6.2%) and MSC-CM (2 w: 27.2 ± 4.9%, 3 w: 44.3 ± 6.9%) groups were also higher than that in the defect (2 w: 3.6 ± 0.6%, 3 w: 5.5 ± 0.9%), PBS (2 w: 15.4 ± 4.5%, 3 w: 23.6 ± 6.5%) and, Exo-antiVEGF groups (2 w: 8.7 ± 1.1%, 3 w: 10.9 ± 1.0%). The newly formed bones at 1–3 weeks after implantation in the Exo-antiVEGF group were significantly decreased in terms of size, compared with the MSC-Exo group (*p* < 0.01). This indicated that the anti-VEGF antibody affected bone formation at an early stage. Four weeks after implantation, the newly formed bone covered almost half of the defect in the MSC-Exo group (48.9 ± 6.2%), and a wide range of the defect area was occupied with the newly formed bone in the MSC-CM group (56.6 ± 6.0%). On the other hand, newly formed bones were not sufficiently found in the defect (7.0 ± 0.9%), PBS (27.3 ± 7.7%), and Exo-antiVEGF (13.4 ± 1.5%) groups. The newly formed bone area in the MSC-Exo group was significantly higher than that in the above mentioned three groups (*p* < 0.01). Moreover, the Exo-antiVEGF group significantly decreased bone formation when compared to the PBS group.

**Fig 5:**
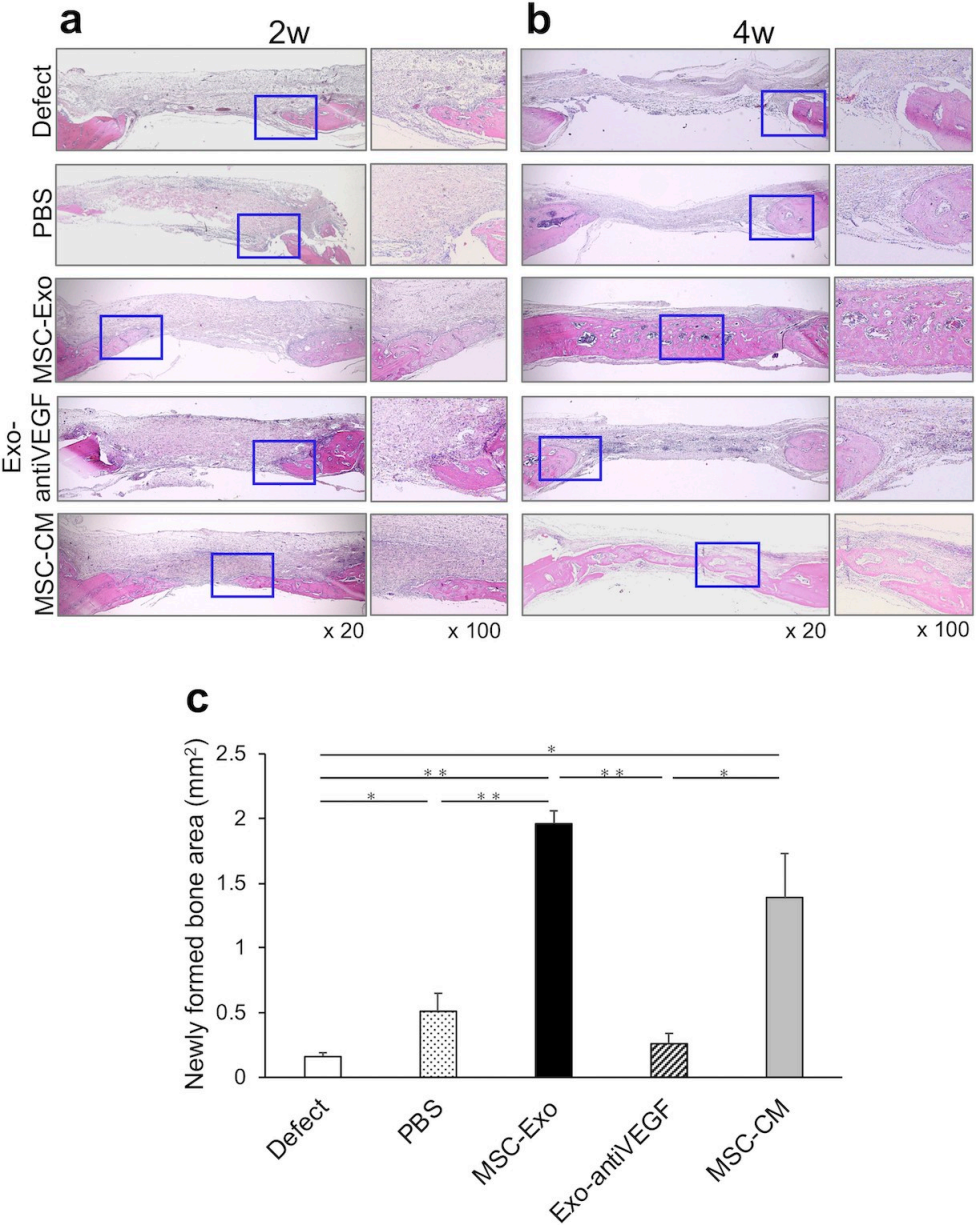
Histological analysis of the newly formed bone. The undecalcified specimens were stained with hematoxylin-eosin. (a) The coronal cross sections of calvarial bone defect 2 weeks after implantation. The immature new bones were observed in the MSC-Exo and MSC-CM groups. (b) The coronal cross sections of calvarial bone defect 4 weeks after implantation. The bone bridge was observed in the MSC-Exo and MSC-CM groups. (c) The newly formed bone area at the coronal cross section (mm^2^). The newly formed bone area was significantly larger in the MSC-Exo group compared to the defect, PBS, and Exo-antiVEGF groups. (n = 5 per group. *, *p* < 0.05; **, *p* < 0.01).

Furthermore, histological findings also showed that the undecalcified specimens in the MSC-Exo group had a larger area of new bone formation compared with that in the defect, PBS, and Exo-antiVEGF groups (Fig 5B). Two weeks after implantation, accumulation of osteoblast-like cells and infiltration of neutrophils were observed in the bone defect margin, and immature trabecular bones were formed along with the osteoid in the MSC-Exo group. Four weeks after implantation, the bone bridge was observed clearly in the MSC-Exo group. Several osteocytes could be observed in the bone bridge, which indicated that the newly formed bone was maturing. In addition, cells were accumulated in the newly formed bone edge, and vasculature structures were observed around it. These results showed the same trend as the results of Micro-CT analysis, and further support the findings of bone formation. Moreover, the newly formed bone areas at the coronal cross section were measured in square millimeters. The newly formed bone areas were 0.16 ± 0.03 mm^2^, 0.51 ± 0.14 mm^2^, 1.96 ± 0.1 mm^2^, 1.39 ± 0.34 mm^2^, and 0.26 ± 0.08 mm^2^ in the defect, PBS, MSC-Exo, MSC-CM, and Exo-antiVEGF groups, respectively (Fig 5C). The newly formed bone area in the MSC-Exo group was significantly larger compared to that in the defect, PBS, and Exo-antiVEGF groups.

### Immunohistochemical staining of the newly formed bone

**Fig 6:**
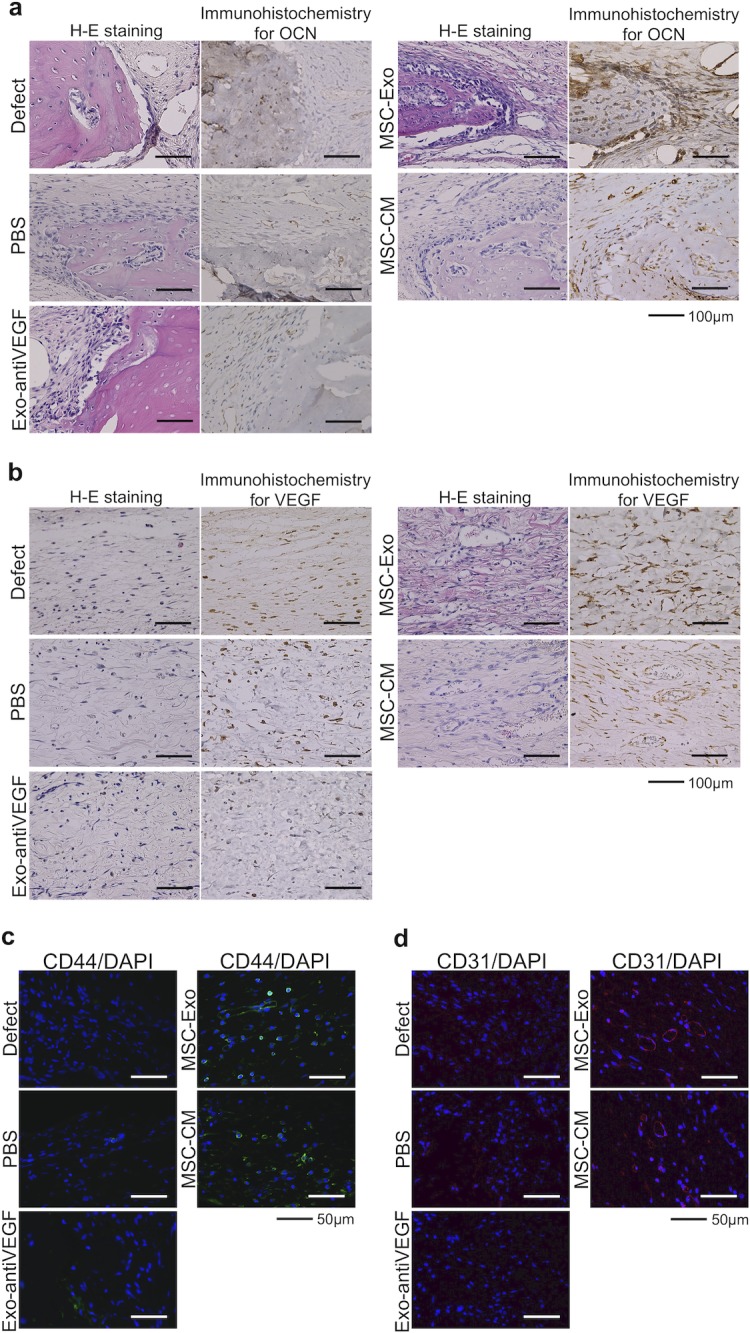
Immunohistochemical analysis of osteogenesis, angiogenesis, rat stem cells, and vascular endothelial cells. (a) The immunohistochemical staining and hematoxylin-eosin (H-E) staining showed that osteoblast-like cells around the edge of the newly formed bone were stained OCN-positive brown in the MSC-Exo group, while there were no marked findings in the defect, PBS, and Exo-antiVEGF groups. (b) The immunohistochemical staining and H-E staining showed that the VEGF-positive brown staining was apparent in vascular endothelial cells at the center area of the whole defect in the MSC-Exo and MSC-CM groups. Left side: H-E staining, right side: immunohistochemical staining. Scale bar: 100 μm. (c) The CD44-positive green-stained cells were clearly observed in the MSC-Exo group. (d) The CD31-positive red-stained cells were observed, as well as the vascular structures in the MSC-Exo group. Scale bar: 50 μm.

The osteogenic marker OCN and the angiogenic marker VEGF were detected using immunohistochemical staining, with results showing that there was no obvious positive staining for OCN in the defect, PBS, and Exo-antiVEGF groups, but positive brown staining for OCN was apparent in the MSC-Exo group (Fig 6A). Moreover, VEGF-positive brown staining was clear in the vascular endothelial cells at the center area of whole defects in the MSC-Exo and MSC-CM groups, while there were no marked findings in the defect, PBS, and Exo-antiVEGF groups (Fig 6B). The number of OCN and VEGF positive stained cells were counted and averaged from 3 random 100 μm^2^ fields around the edge of the newly formed bone (S2 Fig).

Immunohistochemical analysis of CD44 was performed to confirm the mobilization of endogenous MSC to rat calvarial defects by MSC-Exo. CD44 was used to detect rat MSCs in the newly formed bone area of specimens 2 weeks after implantation. The CD44-positive cells were observed around the edge of the newly formed bone in the MSC-Exo and MSC-CM groups (Fig 6C). Similarly, CD31 was used to detect vascular endothelial cells. The CD31-positive cells were also clearly observed around the newly formed bone area in the MSC-Exo and MSC-CM groups, indicating that MSC-Exo and MSC-CM enhanced endogenous stem cell migration and angiogenesis in the rat calvarial bone defect (Fig 6D).

## Discussion

Remarkable progress has been made in the development of regenerative medicine, in terms of creating substitutes for conventional therapies. Although cell-based regenerative medicine is widely studied, its success depends on the viability and activity of stem cells and the surrounding environment of the recipient sites [34]. Stem cells are present in various organs and tissues, and they play an important role in maintaining homeostasis in the healthy body [35]. Thus, the stem cell niche provides a source of quiescent stem cells in response to body conditions such as injury or inflammation [36]. Specifically, some events occur as a stem cell niche response, including secretion of growth factors, migration of stem cells to an inflammation site, differentiation into appropriate cell types, and tissue repair [37]. In cell transplantation, even if only healthy cells are transplanted, the cell viability is low and the regenerative therapeutic effect is not always favorable in the case of a poor surrounding environment due to tissue damage [38].

Our previous studies reported that MSC-CM contain numerous growth factors and investigated the biological effects of MSC-CM that promoted bone regeneration [18]. We showed that MSC-CM enhanced cell migration, expression of angiogenic and osteogenic genes including *VEGF*, *ANG1*, *ANG2*, *COLI*, *OCN*, and Runt-related transcription factor 2 (*Runx2*), and capillary sprout and tube formation of human umbilical cord vein endothelial cells (HUVECs) [28]. *In vivo*, Ogata *et al*. showed that MSC-CM induced early bone regeneration by accelerating migration of stem cells by observing the migration of rat MSCs toward the local area where MSC-CM was implanted using an *in vivo* imaging method [39]. Moreover, it was interesting to note that transplantation of MSC-CM promoted bone regeneration compared to transplantation of MSCs themselves [18]. Thus, we revealed that bone regeneration was mainly promoted by cytokines secreted from MSCs, even without cell transplantation. As mentioned above, MSC-CM contains abundant growth factors that contribute to bone regeneration, but it is also necessary to search for other ingredients in order to clarify the paracrine mechanisms of action of MSC-CM.

Angiogenesis contributes to the progression of osteogenesis, where blood supply induces the migration of osteoblasts and mineralization of bone tissue. Since vascular endothelial cells stimulate osteoblast maturation and activity, angiogenesis is closely associated with successful bone regeneration [40]. Paino *et al*. reported that human dental pulp stem cells had the angiogenic potential and fabricated the vascularized woven bone tissue without scaffolds [41]. VEGF, which is a specific growth factor that acts on vascular endothelial cells, promotes revascularization, regulation of vascular endothelial cell migration, proliferation, and capillary production, and improves the cellular activity of osteoblasts, thereby promoting bone regeneration [42, 43]. Local application of VEGF enhances angiogenesis at defect sites, and promotes osteogenesis [44, 45]. For example, Kumar *et al*. showed that VEGF increased bone mineral density and bone mineral content by increasing cell recruitment using a mouse tibia model [44]. Also, Katagiri *et al*. revealed that the addition of anti-VEGF to MSC-CM reduced blood vessel formation at the bone defect site and resulted in insufficient bone regeneration using a rat calvaria model [21]. Many studies have reported the effect of VEGF on bone regeneration. Local VEGF delivery may couple angiogenesis with bone regeneration and remodeling. In addition, VEGF may act as a central mediator for other factors [45]. Here, the aim of this study was to investigate the effects of MSC-Exo on bone regeneration, particularly with respect to angiogenesis. In our results, MSC-Exo enhanced the expression of not only osteogenesis-related genes but also angiogenesis-related genes such as VEGF, ANG 1, and ANG2, whereas expressions of these genes were decreased in the Exo-antiVEGF group *in vitro*. This indicated that MSC-Exo had an angiogenic potential. In addition, we also confirmed that MSC-Exo had the ability to enhance cell migration. This was also thought to be a crucial step in tissue regeneration by MSC-Exo. Indeed, obvious bone formation was observed *in vivo* in the MSC-Exo group at an early stage (31.5 ± 6.5% at 2 weeks), and the newly formed bone in the MSC-Exo group (48.9 ± 6.2%) was similar to the results in the MSC-CM group (56.6 ± 6.0%) 4 weeks after implantation. Interestingly, histological analysis revealed that the newly formed bone area of the coronal cross section was larger in the MSC-Exo group (1.96 ± 0.1 mm^2^) than in the MSC-CM group (1.39 ± 0.34 mm^2^). Stem cells were found to be accumulated around the bone defects and newly formed bone in the MSC-Exo and MSC-CM groups. Moreover, VEGF was highly expressed in vascular endothelial cells in the MSC-Exo group, while there were no such findings in the defect, PBS, and Exo-antiVEGF groups. Vascular endothelial cells and blood vessels were also observed well around the newly formed bone in the MSC-Exo group following immunofluorescence staining for CD31. These results indicated that MSC-Exo give the recipient cells some information related to angiogenesis and/or bone regeneration, which leads the recipient cells to activate the secretion of VEGF and osteogenesis-related paracrine factors. Therefore, MSC-Exo may partially contribute to controlling the cell niche in the local environment and activating the paracrine factors including VEGF in bone regeneration.

Exosomes contain miRNA and mediate cell-cell communication by transporting genetic information. Also, miRNAs are involved in gene regulatory networks in various signaling pathways and have been studied as target gene therapy agents [46]. Qin *et al*. showed that extracellular vesicles secreted from MSCs promoted bone regeneration *in vivo*, and three osteogenesis-related miRNAs (miR-196a, miR-27a, and miR-206) in extracellular vesicles were highly upregulated, and also that miR-196a was considered one of the most important regulators of osteogenesis [47]. Furuta *et al*. showed that the differentially expressed miRNA such as miR-21, miR-4532, miR-125b-5p, and miR-338-3p in exosomes secreted from MSCs might contribute to the enhancement of osteogenesis and angiogenesis [48]. Chen *et al*. revealed that exosomes derived from miR-375-overexpressing human adipose mesenchymal stem cells had osteogenic potential and promoted bone regeneration [49]. Therefore, early bone regeneration induced by exosomes was considered to depend on the regulation of multiple miRNAs and their relevant pathways, and miRNAs affect tissue development and homeostasis by regulating gene expression. In our study, we found that MSC-Exo contain miRNAs that may enhance VEGF secretion from the recipient cells, which contributes to bone regeneration.

Several factors contained in MSC-CM that contribute to bone regeneration have been investigated so far, but MSC-CM also contains other miscellaneous components that have not been fully elucidated. To partially address this, we isolated MSC-Exo, which was a single component type in MSC-CM, and found that it promoted bone regeneration by enhancing angiogenesis. Recent studies have reported that exosomes have various potential applications in diagnostics and treatment [50, 51, 52]. The application of exosomes could help to avoid the potential risks of toxicity and immunogenicity caused by biomaterial processing [53]. In addition, exosomes have a lower risk of serious complications such as tumorigenesis or emboli formation when compared to cell transplantation [54]. Hence, exosomes are suggested as a potential regenerative agent that can replace the conventional methods of bone regeneration including stem cell transplantation. However, further studies are needed to verify the active molecules of exosomes in angiogenesis, cell migration, and bone regeneration under various cell environments. It is important to identify the optimal conditions for exosomes to exert the most effective action on bone regeneration, potentially contributing to the basis of drug discovery, offering novel insights into cell-based regenerative medicine that can be applied in various medical fields.

## Conclusion

Our results suggest that MSC-Exo have a remarkable osteogenic potential, as we reported previously in MSC-CM. Exosomes contained in MSC-CM may play important roles in bone regeneration by enhancing angiogenesis. Our findings also indicate that MSC-Exo can be used as bioactive agents for bone regeneration.

## Supporting information

S1 Fig Images of migrated cells. The hMSCs that passed through the membrane were in focus clearly. The migrated cell number in the MSC-Exo group was higher than that in the DMEM(-) and Exo-antiVEGF groups.
(TIFF)

S2 Fig The number of OCN and VEGF positive stained cells. The positive stained cells were counted and averaged from 3 random 100 μm^2^ fields. The numbers of OCN and VEGF positive stained cells in the MSC-Exo group were higher than that in the DMEM(-) and Exo-antiVEGF groups. (n = 3 per group. *, *p* < 0.05; **, *p* < 0.01).
(TIFF)
