# Supplementary figures and images for: Exosomes from conditioned media of bone marrow-derived mesenchymal stem cells promote bone regeneration by enhancing angiogenesis
Source: PLoS One. 2019 Nov 21;14(11):e0225472. doi: 10.1371/journal.pone.0225472 (PMC6872157; doi:10.1371/journal.pone.0225472)

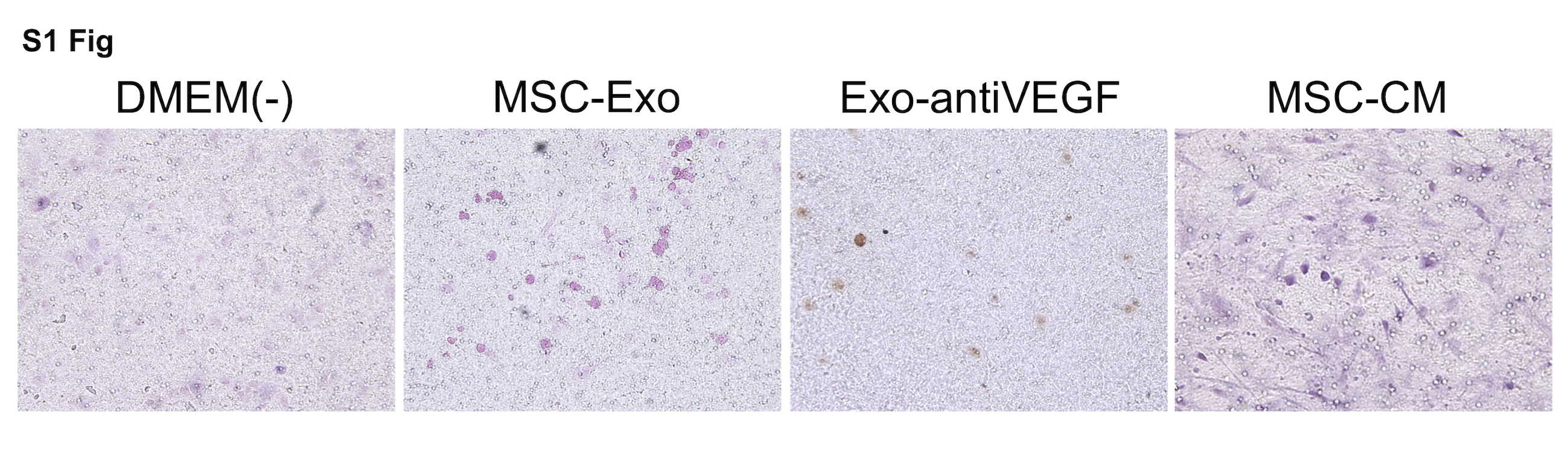

Supplement: S1 Fig — The hMSCs that passed through the membrane were in focus clearly. The migrated cell number in the MSC-Exo group was higher than that in the DMEM(-) and Exo-antiVEGF groups. (TIFF) [file pone.0225472.s001.tiff]

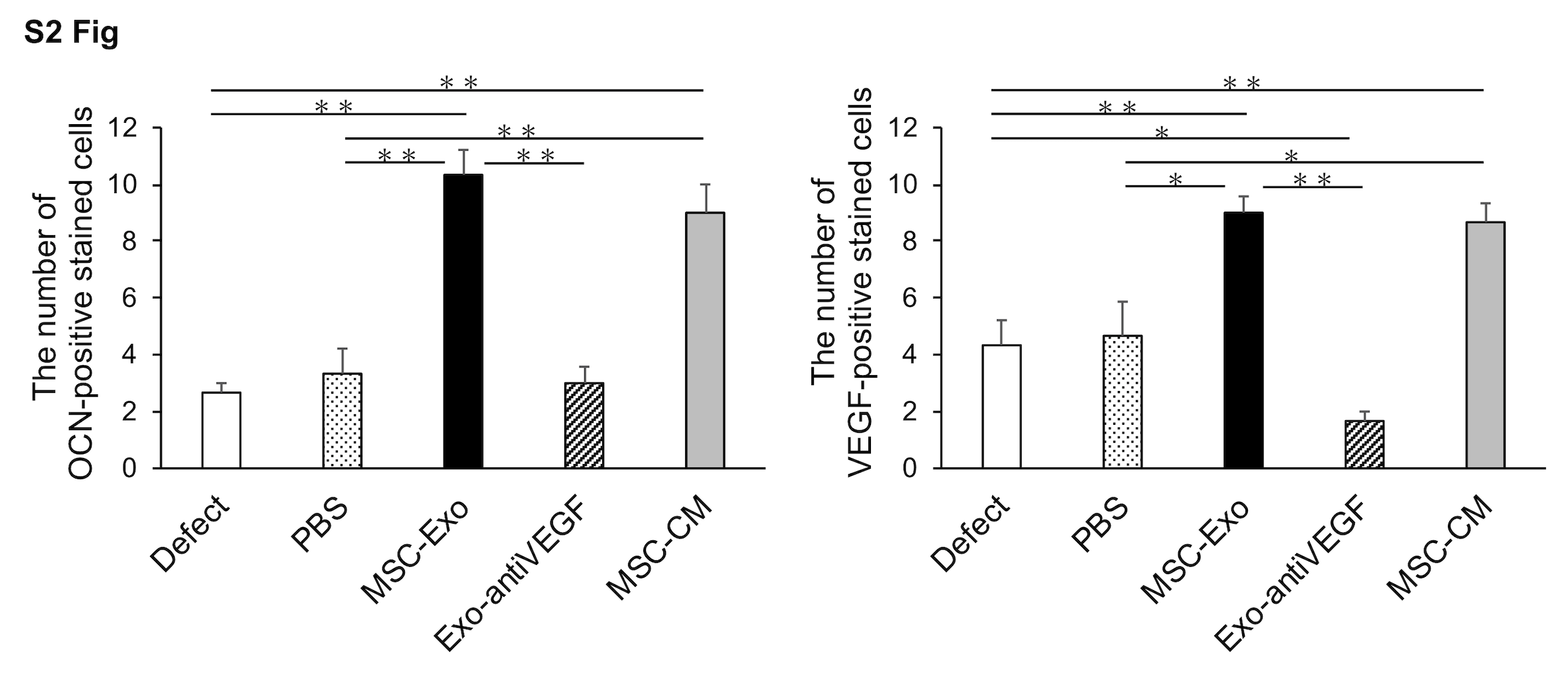

Supplement: S2 Fig — The positive stained cells were counted and averaged from 3 random 100 μm2 fields. The numbers of OCN and VEGF positive stained cells in the MSC-Exo group were higher than that in the DMEM(-) and Exo-antiVEGF groups. (n = 3 per group. *, p < 0.05; **, p < 0.01). (TIFF) [file pone.0225472.s002.tiff]
